# A Novel Scleral Tunnel Technique for the Prevention of Ahmed Glaucoma Valve Tube Exposure

**DOI:** 10.7759/cureus.79290

**Published:** 2025-02-19

**Authors:** Yusaku Miura, Ken Fukuda, Kenji Yamashiro

**Affiliations:** 1 Ophthalmology and Visual Science, Kochi Medical School, Nankoku, JPN

**Keywords:** ahmed glaucoma valve, glaucoma, glaucoma drainage device, scleral tunnel, tube exposure

## Abstract

Tube exposure after Ahmed glaucoma valve (AGV) implantation is a serious complication in eyes with glaucoma. This study aimed to present an effective and minimally invasive scleral tunnel technique for preventing tube exposure. A scleral tunnel was created using a 22-gauge needle between two scleral incisions. The scleral incisions were made 2 mm and 8 mm from the limbus for a simple AVG implantation, and 4 mm and 8 mm from the limbus for an AVG implantation performed together with pars plana vitrectomy (PPV), in which a trocar for the PPV was inserted through the scleral incision at the 4 mm position. After trimming the tube to an appropriate length, it was inserted into the scleral tunnel created by the 22-gauge needle. The tube was then removed from the sclera, and its tip was inserted into the posterior chamber through the scleral incision. After confirming the insertion of the tube into the posterior chamber, the Tenon’s capsule and conjunctiva were sutured using 8-0 Vicryl sutures. No postoperative complications, including tube exposure, were observed. This novel technique is an effective and minimally invasive method for preventing tube exposure after AGV implantation.

## Introduction

A glaucoma drainage device (GDD) is used to reduce intraocular pressure (IOP) by filtering the aqueous humor through a tube inserted intraocularly into a plate fixed to the sclera. Although it is an effective treatment option in cases of refractory glaucoma and unsuccessful trabeculectomy (TLE), there is a possibility of tube exposure, a complication unique to GDD. If the tube is covered only by the conjunctiva, it may lead to erosion of the conjunctiva followed by tube exposure. The causes of tube exposure are believed to be mechanical factors such as excessive tension on the tube, frequent trauma due to blinking of the eyelids, inflammation, and ischemic pathogenesis due to compression of the conjunctival blood vessels [1,2]. Therefore, it is necessary to cover the tube intraoperatively with a patch graft or autologous sclera to avoid tube exposure.

Materials for patch grafts include the donor sclera, cornea, femoral fascia, dura mater, and pericardium. However, the use of patch grafts carries the risk of viral infection and rejection reactions. Additionally, preoperative preparation of the grafts may be required, and they can be costly and difficult to obtain [3]. Moreover, if the patch graft is not sutured properly or is too thick, there is a risk of conjunctival erosion due to constant rubbing of the eyelids against its raised surface.

Alternative methods for covering the tube, such as autologous half-thickness scleral flaps [4-9] or autologous scleral tunnels [7,8,10,11], are less costly and eliminate the risk of viral infection or rejection. Previous studies have shown no significant differences in the incidence of tube exposure between autologous scleral and patch grafts [7]. Some studies have reported that autologous scleral grafts are superior to patch grafts for reducing tube exposure [12,13] and that using autologous sclera to prevent tube exposure might be beneficial. However, there are numerous methods of covering the tube with autologous sclera, and no definitive conclusion has been reached regarding the optimal method.

In this study, we describe a new surgical technique for burying the tube within the sclera using a scleral tunnel created with a 22-gauge needle to prevent exposure of the tube of Ahmed glaucoma valve (AGV), a type of GDD.

## Case presentation

Case 1

An 88-year-old man presented with open-angle glaucoma of the left eye. He had previously undergone cataract extraction, intraocular lens (IOL) implantation, and penetrating keratoplasty (PKP) for corneal endothelial damage in the left eye. Preoperative best corrected visual acuity (BCVA) was 0.2 in the left eye. Goldmann applanation tonometry revealed an IOP of 25 mmHg in the left eye while using glaucoma eye drops (carteolol/latanoprost fixed combination). Visual field examination with Goldmann perimetry showed central vision field loss in III/4e. Slit-lamp examination revealed slight edema in the donor cornea after PKP, while the upper conjunctiva appeared normal. As IOP reduction in the left eye was inadequate, AGV implantation (tube insertion into the ciliary sulcus) was performed.

**Figure 1:**
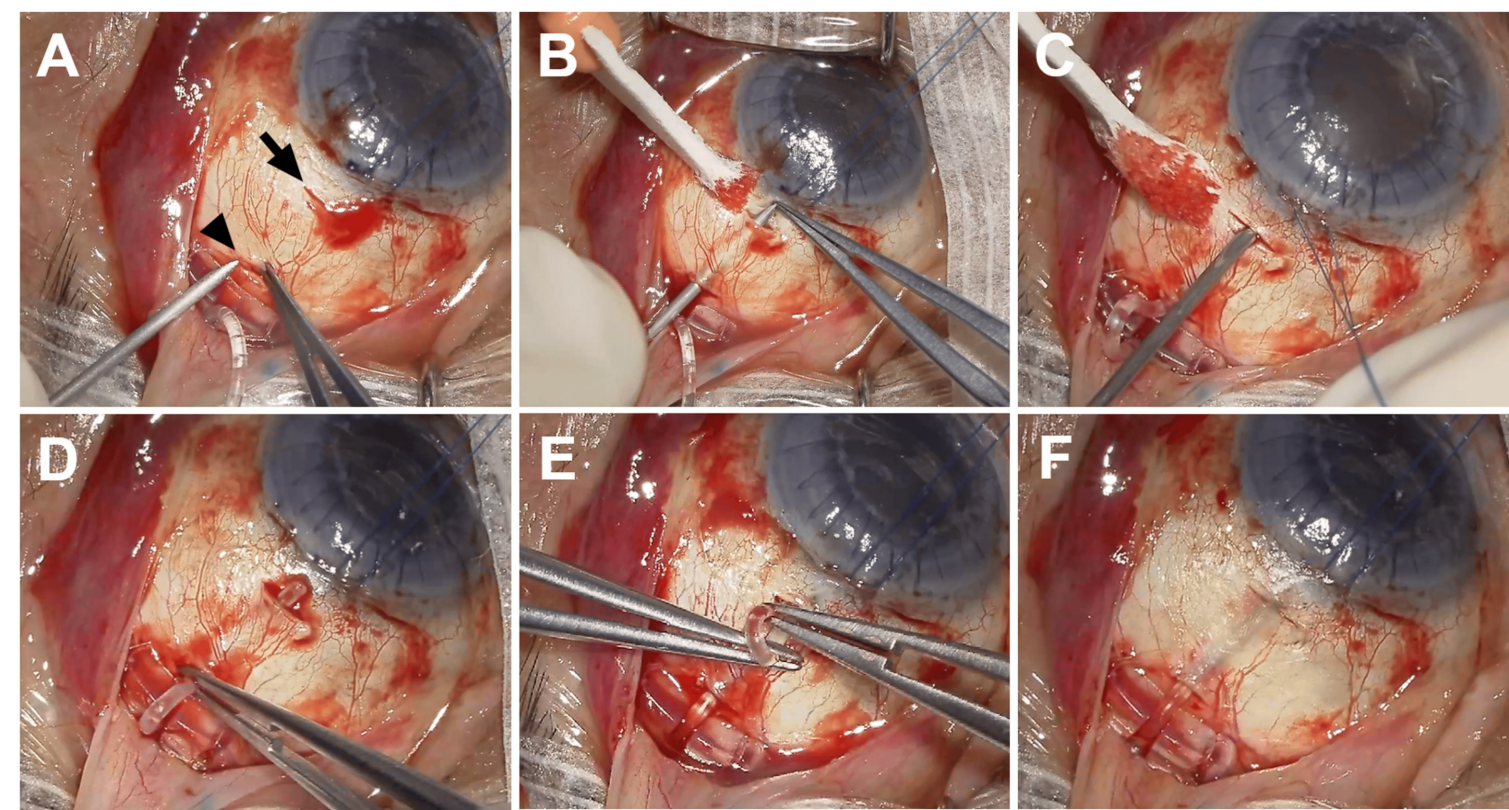
Ahmed glaucoma valve (AGV) tube insertion into the ciliary sulcus from the surgeon’s view. A: An approximately 1-mm-wide scleral incision was made using a microsurgical blade, 2 mm (arrow) and 8 mm (arrowhead) from the limbus. B: A scleral tunnel was created using a 22-gauge needle inserted from the scleral incision at the 8 mm position, bevel-down, toward the scleral incision at the 2 mm position, carefully avoiding perforating the sclera. C: A 22-gauge needle was inserted at the 2 mm position into the posterior chamber parallel to the iris after injection of viscoelastic material through the side ports at the limbus. D: The tube was inserted into the scleral tunnel created using the 22-gauge needle after trimming the tube to an appropriate length. E: The tip of the tube was inserted into the posterior chamber through the scleral incision after pulling the tube out of the sclera at the 2 mm position. F: The insertion of the tube into the posterior chamber was confirmed.

After peribulbar anesthesia using a sub-Tenon injection of 2% lidocaine, the AGV was fixed to the sclera in the superotemporal quadrant, 9 mm from the corneal limbus with 7-0 nylon sutures. An approximately 1-mm-wide scleral incision was made using a microsurgical blade 2 and 8 mm from the limbus (Figure 1). Subsequently, a 22-gauge needle was inserted from the scleral incision at the 8 mm position, bevel-down, toward the scleral incision at the 2 mm position, while carefully avoiding perforating the sclera, to create a scleral tunnel. Side ports were created at the limbus and viscoelastic material was injected into the posterior chamber. A 22-gauge needle was then inserted through the scleral incision at the 2 mm position into the posterior chamber parallel to the iris. After trimming the tube to an appropriate length, it was inserted into the scleral tunnel created by the 22-gauge needle. After pulling the tube out of the sclera at the 2 mm position, the tip of the tube was inserted into the posterior chamber through the scleral incision. After confirming the insertion of the tube into the posterior chamber, the viscoelastic material was flushed out with a balanced salt solution, and Tenon’s capsule and conjunctiva were sutured with 8-0 Vicryl sutures.

There were no intraoperative complications. The patient was treated postoperatively with 0.5% moxifloxacin eye drop four times a day and 0.1% betamethasone sodium phosphate eye drop six times a day. At 15 months postoperatively, the left IOP was 9 mmHg without glaucoma eye drops. There were no postoperative complications, including tube exposure.

Case 2

A 70-year-old man developed neovascular glaucoma of the right eye. He had previously undergone cataract extraction, IOL implantation, TLE, and pars plana vitrectomy (PPV) for bleb infection in the right eye. BCVA was 0.04 in the right eye. Goldmann applanation tonometry showed an IOP of 32 mmHg in the right eye with glaucoma eye drops (latanoprost, brimonidine, and ripasudil). Visual field examination with Goldmann perimetry revealed central visual field loss and only a slight visual field in the right inferior quadrant at I/4. Slit-lamp examination demonstrated a flat avascular bleb in the upper part, with no signs of infection. Because the IOP reduction in the right eye was inadequate, AGV implantation (tube insertion into the vitreous cavity) and PPV were performed.

**Figure 2:**
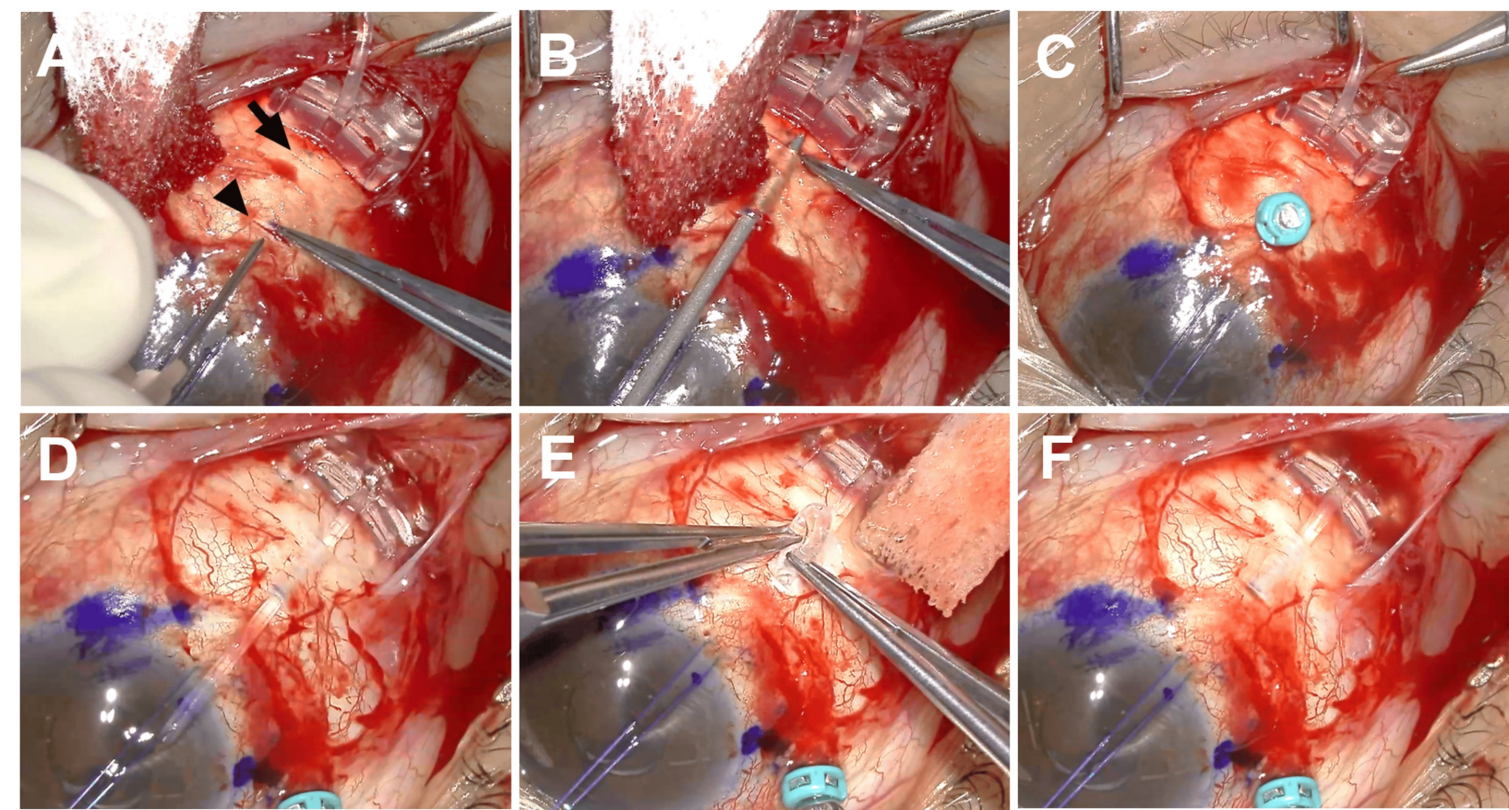
Ahmed glaucoma valve (AGV) tube insertion into the vitreous cavity from the surgeon’s view. A: An approximately 1-mm-wide scleral incision was made using a microsurgical blade, 4 mm (arrowhead) and 8 mm (arrow) from the limbus. B: A scleral tunnel was created using a 22-gauge needle inserted from the scleral incision at the 4 mm position, bevel-down, toward the scleral incision at the 8 mm position, carefully avoiding perforating the sclera. C: A 25-gauge trocar was inserted through the scleral incision at the 4 mm position and the residual vitreous was removed by pars plana vitrectomy. D: The tube was inserted into the scleral tunnel created using the 22-gauge needle after removing the trocar at the 4 mm position. E: The tip of the tube was inserted into the vitreous cavity through the scleral incision after pulling the tube out of the sclera at the 4 mm position. F: The insertion of the tube into the vitreous cavity was confirmed.

After peribulbar anesthesia using a sub-Tenon injection of 2% lidocaine, the AGV was fixed to the sclera in the inferotemporal quadrant, 9 mm from the corneal limbus with 7-0 nylon sutures. An approximately 1-mm-wide scleral incision was made using a microsurgical blade, 4 mm and 8 mm from the limbus (Figure 2). Subsequently, a 22-gauge needle was inserted from the scleral incision at the 4 mm position, bevel-down, toward the scleral incision at the 8 mm position, while carefully avoiding perforating the sclera to create a scleral tunnel. A 25-gauge trocar was then inserted through the scleral incision at the 4 mm position. The tube was trimmed to an appropriate length after PPV to remove the residual vitreous. Next, the trocar at the 4 mm position was removed, and the tube was inserted into the scleral tunnel created by the 22-gauge needle. After pulling the tube out of the scleral incision at the 4 mm position, the tip of the tube was inserted into the vitreous cavity through the scleral incision created by the 25-gauge trocar. After confirming the insertion of the tube into the vitreous cavity, all trocars were removed, and Tenon’s capsule and conjunctiva were sutured with 8-0 Vicryl sutures.

There were no intraoperative complications. The patient was treated postoperatively with 0.5% moxifloxacin eye drop four times a day and 0.1% betamethasone sodium phosphate eye drop four times a day. At 21 months postoperatively, the right IOP was 21 mmHg with glaucoma eye drops (tafluprost, brimonidine/timolol fixed combination). There were no postoperative complications, including tube exposure.

## Discussion

Despite the fact that the use of patch grafts or autologous sclera to cover the tube is considered an essential technique for preventing tube exposure after AGV implantation, tube exposure still occurs in 4.3% to 14.3% of cases [3,14-16]. The causes of tube exposure are diverse, but it is believed that friction between the conjunctiva, sclera covering the tube, and eyelids due to eye movements and blinking may lead to the thinning of these tissues [17]. Tube exposure is more likely when the tube is flexible or mobile [18-20]. Therefore, to prevent tube exposure, it is essential to minimize the flexibility and mobility of the tube, which necessitates secure fixation of the tube or patch graft to the sclera with nylon suture. Nevertheless, in previous reports, fixation of the tube to the sclera appeared to be inadequate compared to our technique. Previous studies used a crescent knife to create a scleral tunnel; however, this technique can create gaps between the scleral tunnel and tube, making complete fixation of the tube difficult [8,10-12]. Furthermore, when the tube is sutured to the sclera with nylon sutures, it involves anchoring the tube with individual sutures, and it may not be sufficient to prevent the flexibility and mobility of the tube. The outer diameter of the AGV tube is approximately 0.63 mm, whereas the outer diameter of a 22-gauge needle is approximately 0.71 mm. When using our technique of creating a scleral tunnel with a 22-gauge needle, the gap between the lumen of the scleral tunnel and the inserted tube is minimal. Furthermore, burying the entire tube within the long scleral tunnel allowed complete fixation within the scleral tunnel. Consequently, the tube has minimal flexibility and mobility, which may be helpful in preventing tube exposure.

Additionally, it has been speculated that while covering the tube with autologous sclera, if the blood flow to the sclera over the tube is maintained, thinning of the sclera is less likely [10]. The creation of an autologous scleral flap typically requires three scleral incisions of 3-10 mm [4-9]. However, in our technique, we only made scleral incisions of less than 1 mm on both the proximal and distal sides, significantly reducing the extent of the scleral incision. This small incision range allows sufficient blood flow to the sclera over the tube, which may help in preventing scleral thinning.

Furthermore, the autologous scleral flap technique is complicated due to the need for flap creation and suturing. Additionally, the previous scleral tunnel methods required expensive instruments such as a crescent knife to create a scleral tunnel or disposable vitreous forceps to insert the tube into the vitreous cavity [8,10-12]. In contrast, our method involves creating a scleral tunnel using only a 22-gauge needle, which simplifies the procedure and reduces surgical time. Moreover, because we used only a microsurgical blade and 22-gauge needle, the costs were low. Since our procedure does not require sutures, we can avoid the complications associated with sutures, such as suture degradation leading to breakage, erosion of the conjunctiva or sclera at the suture ligature, and scleral perforation from needle passage.

In patients in whom we used our scleral tunnel technique using a 22-gauge needle, no intraoperative and postoperative complications were observed. However, our technique has the potential for several complications. For example, since there is a slight gap between the AGV tube and the scleral tunnel, peritubal leakage or associated hypotony may occur. Additionally, while inserting the 22G needle, there is a risk of perforating the sclera or choroid. To avoid this, it is necessary to insert the needle while maintaining a consistent insertion depth and keeping it visible through the surface of the sclera. It is also effective to press down on the scleral surface with a forceps or cotton swab to flatten the curve of the scleral surface while inserting the needle to maintain a consistent insertion depth. Furthermore, it is crucial to insert the 22G needle with the bevel-down to avoid choroidal perforation, which may lead to serious complications. We believe that a bevel-down needle has a significantly lower risk of choroidal perforation compared to a bevel-up needle. Moreover, multiple needle tracks or repeated manipulations may cause damage to the scleral tunnel lumen, potentially making AGV tube insertion difficult. Therefore, when creating the scleral tunnel, the 22G needle should be inserted in a single continuous motion without withdrawal and reinsertion. Lastly, since we have no experience, it is unclear whether the insertion of the AGV tube into the anterior chamber using our technique is effective.

## Conclusions

The technique described here has some advantages over previous methods, including reducing the flexibility and mobility of the tube, maintaining the blood flow to the sclera over the tube, lower cost, and simpler procedure. However, due to the small sample size, the short follow-up period of this study, and the potential for some complications, we could not confirm the efficacy and safety of this new technique, and larger-scale studies with longer follow-up periods are required.
